# Impacts of methyl jasmonate treatments on quality properties and bioactive compounds of jujube fruit (*Ziziphus jujuba*) during cold storage

**DOI:** 10.1186/s12870-025-07140-2

**Published:** 2025-08-11

**Authors:** Burhan Ozturk, Orhan Karakaya, Erdal Aglar, Mehmet Utku Hancı, Ahmet Ozturk, Mohammadreza Asghari

**Affiliations:** 1 Faculty of Agriculture, Department of Horticulture, Ordu University, Ordu, Türkiye; 2 Faculty of Agriculture, Department of Horticulture, Sakarya University of Applied Sciences, Sakarya, Türkiye; 3 Faculty of Arts, Design and Architecture, Department of Landscape Architecture, Munzur University, Tunceli, Türkiye; 4 Faculty of Agriculture, Department of Horticulture, Ondokuz Mayıs University, Samsun, Türkiye; 5 Faculty of Agriculture, Department of Horticulture, Urmia University, Urmia, Iran

**Keywords:** Antioxidant, Color, Flavonoids, Phenolics, Respiration rate, Weight loss

## Abstract

**Background:**

Jujube is an important fruit species due to its high nutritional value and health benefits. However, the short shelf life of fruit causes quality losses, making it difficult to trade. This study was conducted to determine the effects of postharvest methyl jasmonate (MeJA) applications on the fruit quality and biochemical properties of ‘Li’ jujube (*Ziziphus jujuba* Mill.) cultivar. In the study, fruits were pre-cooled at 7 °C and 85 ± 5% humidity for 24 h and those with diseases, pests, and mechanical damage were eliminated. Then, MeJA was applied to the fruits at concentrations of 0.2 mM and 0.4 mM and stored at 0 ± 0.5 °C and 90 ± 5% humidity for 45 days and analyzes were performed at 15-day intervals.

**Results:**

This study showed that MeJA applications positively affected the postharvest quality of jujube fruit. MeJA-applied fruits exhibited lower weight loss and lower respiration rates, and MeJA applications helped preserve fruit color and maintained fruit firmness for a longer time. MeJA was effective in maintaining the total phenolic and flavonoid content in fruit content and the loss of vitamin C content was lower with MeJA application. MeJA was effective for determining soluble solids content (SSC), and titratable acidity and delayed ripening in fruit.

**Conclussion:**

MeJA application increases the commercial value of fruits by reducing weight loss, slowing down the ripening process, and preserving higher nutritional content and antioxidant activities and suggest that it can be used as a feasible method for improving fruit protection strategies. MeJA application stands out as an effective method for extending the shelf life of jujube fruits by preserving their post-harvest quality and nutritional values.

**Supplementary Information:**

The online version contains supplementary material available at 10.1186/s12870-025-07140-2.

## Background

Jujube (*Ziziphus jujuba* Mill) is an important fruit species with a long history of nutritional and medical treatment in different cultures around the world. Jujube offers significant health benefits thanks to the vitamin C, flavonoids, proanthocyanidins, alkaloids, and other antioxidants it contains [1, 2]. Jujube, known for its heart health-supporting, immune system strengthening, and anti-aging properties, also has positive effects on the digestive and respiration systems [3]. However, jujube fruit faces significant quality losses and a short shelf life in the post-harvest period. The fruit tends to deteriorate rapidly after ripening; the adverse physiological processes such as softening, color change, and water loss become more pronounced during storage. These processes significantly reduce the organoleptic quality and nutritional value of fruit. Factors such as jujube’s sensitivity to climatic conditions, increased respiration rate, and oxidative stress also lead to deterioration [4]. Many applications have been reported to extend the shelf life of jujube and reduce quality losses. Physical and chemical interventions such as modified atmosphere packaging, edible coating applications, and cold storage are among the main techniques used to preserve fruit quality [5, 6]. However, phytohormone applications play an important role in improving the quality and shelf life of jujube. Methyl jasmonate is an important phytohormone that activates the defense mechanisms and regulates biochemical processes [7]. This hormone helps prevent fruit deterioration by reducing oxidative stress in fruit tissues. This mechanism increases the effectiveness of methyl jasmonate in fruit quality preservation. Methyl jasmonate is a compound that is widely found in nature and plays important biological roles, especially in plants. This compound is biosynthesized in plants through a series of chemical reactions that begin with the enzyme lipoxygenase and are derived from linolenic acid. Methyl jasmonate effectively combats diseases and pests, reduces oxidative stress, and prevents cold damage by activating plant responses to stress conditions [8, 9]. Exogen MeJA application can promote plant growth, increase the production of phenolic and volatile compounds, as well as improve overall plant health [10]. The postharvest effects of MeJA are also an important research topic. Previous studies show that postharvest MeJA applications provide significant improvements on the quality parameters of fruits and vegetables. In particular, effects such as controlling fruit ripening, improving color and texture quality, preventing water loss, and increasing antioxidant levels confirm the potential of MeJA to improve post-harvest fruit quality [11]. However, MeJA is effective in extending shelf life by reducing oxidative stress in fruit tissue and preventing spoilage. The question of why post-harvest methyl jasmonate applications are so important stems from the fact that fruits and vegetables become more sensitive to environmental factors, and quality losses are frequently observed during this period. Advantages such as preventing fruit deterioration, managing fruit ripening, and supporting color development increase the effectiveness of methyl jasmonate applications [12, 13]. The aim of the study was to determine the effects of postharvest MeJA applications on jujube (*Ziziphus jujuba* L.) fruit quality and biochemical properties.

## Materials and methods

In this study, the effects of postharvest application of different doses of MeJA on ‘Li’ jujube (*Ziziphus jujuba* Mill.) cultivar fruits were investigated. The fruits used in the study were hand-harvested from trees planted in the north-south direction at 3.5 × 2.0 m intervals and with a central leader training system applied in a jujube orchard in Amasya on October 8, 2016, when the outer color of the fruit peel started to change from yellow/green to brown/reddish. The harvested fruits were transffered to the cold storage facility of Ordu University, Faculty of Agriculture, Department of Horticulture as quickly as possible. First, the fruits were pre-cooled for 24 h in a cold storage at 7 °C and 85 ± 5% relative humidity. After the pre-cooling process was completed, the fruit were selected according to homogeneous dimensions, the disease, harmful or mechanical damaged fruits were extracted. This pre-elimination was performed in order to increase the reliability of the results in experimental applications and to provide standardity between examples. In this study, methyl jasmonate (MeJA) was applied to the chosen jujube fruits at two different concentrations (0.2 mM and 0.4 mM). As a method of application, it was preferred to immerse the fruits into the MeJA solution for 15 min.

The main reason for the determination of the application dose (0.2 mM and 0.4 mM) and duration (15 min) in this way is that these concentrations and durations have been revealed in scientific research on different types of fruit such as apples, pears, peaches, strawberries and apricots. After MeJA application, fruits were dried at room temperature, placed in plastic boxes, and stored in a cold room (0 ± 0.5 °C and 90 ± 5% relative humidity) for storage. The following measurements and analyses were performed at 15-day intervals on fruits stored for 45 days. The study was designed as three repetitions. 15 fruits for each repetition during the measurement periods, 45 fruits for an application and 135 fruits for each measurement period and 540 in total were used.

### Weight loss

At the beginning of cold storage, initial weights (Wi) of the fruits were determined using a digital scale with a precision of 0.01 g (Radwag, Poland). Then, on d 15th, 30th and 45th days of the storage, final weights (Wf) were determined. The weight loss observed in fruits was based on the weight at the beginning of each measurement period and determined as a percentage using the equation given below (Eq. 1).

1 $$WL=\frac{{{w_i} - {w_f}}}{{{w_i}}} \times 100$$

### Respiration rate

Five fruits were placed in airtight containers, and after a 2 h waiting period, the amount of CO_2_ released into the atmosphere was measured using a gas analyzer (Vernier, USA). The respiration rate was expressed as nmol kg^− 1^ s^− 1^. Respiration Rate = (Vj – Vf) x %CO_2_ × 10 / FW x T,

Vj = Jar volume, Vf = Fruit volume, FW = Fruit Weight (kg), T = Time (Hour).

### Fruit flesh firmness

Ten fruit from each replication were used to detect firmness. The measurements were performed by a durometer (France, Agrosta^®^) with a flat cylindrical tip and with a diameter of 4.1 mm. The tip of the durometer was slightly and longitudinally pressed into the outer skin of the fruit and the percentage (%) value on the screen was recorded. If the value was close to 100, the fruit was considered very firm and close to 0 shown extremely soft fruit.

### Fruit color

Fruit color (on ten fruit) was determined in terms of CIE L*, a* and b*. Fruit color was determined from points determined at 2 opposite poles of the equatorial part of the fruit using a colorimeter (Minolta, model CR-400, Tokyo, Japan) in 10 fruits. L *, a * and b * values are used to define the 3-D colour space. Chroma [C*= (a^*2^+b^*2^)^1/2^] and hue angle (h^°^= tan^-1^ b^*^/a^*^) were calculated as per the equations shown in parentheses.

### Soluble solids content, titratable acidity, and vitamin C

Ten fruits in each replication were washed with distilled water. The fruits were homogenized in a blender (Promix HR2653, Philips, Türkiye), and the homogenate was filtered through cheesecloth to obtain a juice filtrate. SSC was determined using a digital refractometer (Atago PAL-1, USA) and recorded as a percentage (%). For TA measurements, 10 mL of distilled water was added to 10 mL of juice. Then 0.1 N sodium hydroxide (NaOH) was added until pH reached 8.2. Based on the amount of NaOH consumed in the titration, TA was calculated and expressed as %. Vitamin C content was determined using a Reflectoquant Plus 10 device (Merck RQflex Plus 10, Türkiye). A 0.5 mL sample of the juice extract was diluted to 5 mL with 0.5% oxalic acid. The ascorbic acid test kit (Catalog No: 116981, Merck, Germany) was briefly immersed in the solution for 2 s, left to oxidize for 8 s, and measured in the device’s test adapter after 15 s. Results were expressed as mg 100 g^-1^.

### Total phenolic content

Approximately 5 fruits from each replication were washed with distilled water, deseeded, and homogenized in a blender. Approximately 30 mL of the homogenized fruit sample was transferred to 50 mL falcon tubes. The fruit samples were centrifuged at 4.0℃, 12,000 rpm for 35 min, separating the pulp and juice. Bioactive compounds were determined from the fruit juice using a UV-Vis spectrophotometer (Shimadzu, Kyoto, Japan). Total phenolic content was detected according to Singleton and Rossi [14], and expressed as g 100 g^-1^ GAE (gallic acid equivalent) fresh weight (fw).

### Total flavonoid content

The total flavonoids content was measured using a modified method reported by Zhishen et al. [15]. A 600 µL fruit extract sample was mixed with 3.7 mL of methanol to make 4.3 mL. Then, 100 µL of 10% aluminum nitrate [Al(NO_3_)_3_] and ammonium acetate (0.1 M) were added to reach a final volume of 4.5 mL. The solution was incubated in the dark for 40 min at room temperature, and absorbance was measured at 415 nm. Results were expressed as g 100 g^-1^ QE (quercetin equivalent) fw.

### Antioxidant activity (DPPH and FRAP)

The DPPH free radical-scavenging activity of the extract obtained from jujube samples was determined by modifying the method of Blois [16]. In the study, DPPH solution was chosen as the free radical. 500 µL of the extract was taken, and 2.5 mL of ethanol was added to make up to 3.0 mL. The total volume of 0.5 ml of 0.1 mM ethanol solution of DPPH free radicals, sample extract, and standard antioxidant solution (50–500 µg/mL) was 4 mL. The solution was mixed by vortex for 1.0 min and placed in a dark environment for 30 min at room conditions. The absorbance of the solution was read at 517 nm in a UV-vis spectrophotometer, and the results were expressed as mmol Trolox equivalent (TE) 100 g^-1^ fw.

For FRAP analysis, phosphate buffer (1.15 mL, 0.2 M, pH 6.7) was first prepared and added to 100 µL of jujube extract sample together with potassium ferricyanide [K_3_Fe(CN)_6_] (1.25 mL, % 1). Then, the reaction mixture was kept at 50 °C for 20 min and then cooled to room temperature. Then, trichloroacetic acid [TCA, (1.25 mL, 10%)] and iron chloride [FeCl3 (0.25 mL, 0.1%)] were added and mixed by vortexing for 1.0 min. The absorbance of the solution was read at 700 nm in a UV-vis spectrophotometer, and the results were expressed in mmol TE 100 g^-1^ fw.

### Statistical analysis

The normal distribution control of the data obtained from the study was done by Kolmogorov-Smirnov, test and the homogeneity control of variances was performed using the Levene test. The descriptive statistics of the data were evaluated using variance analysis. After the data were analyzed with the analysis of variance, the level of significance between treatments was determined by the Tukey multiple comparison test. SAS package program (SAS 9.1 version, USA) was used for statistical analysis. The level of significance was considered α = 5% in the statistical analysis.

## Results and discussion

### Weight loss

**Fig. 1:**
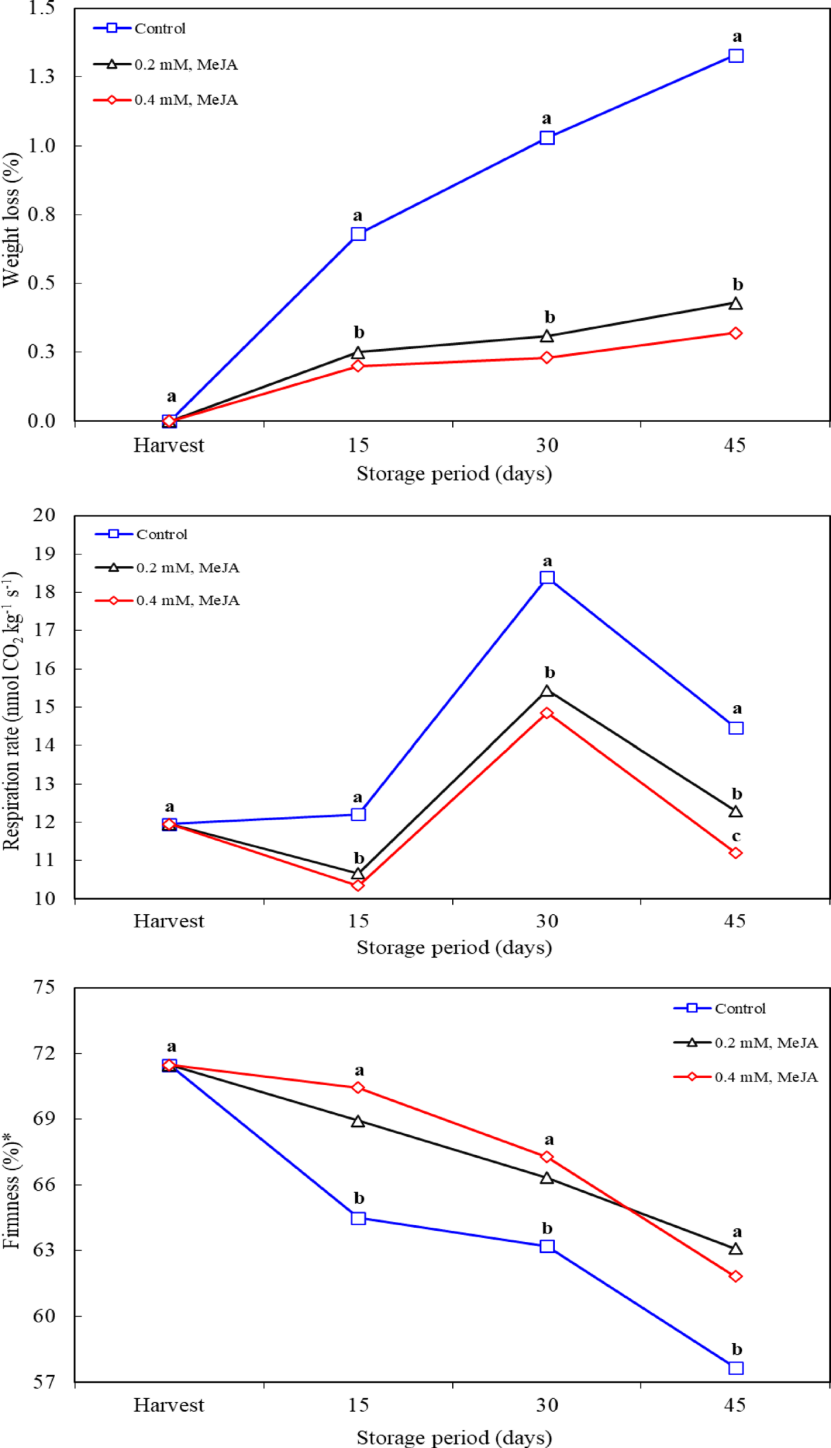
Effects of methyl jasmonate treatments on weight loss, respiration rate and firmness of jujube fruit (*Ziziphus jujuba*) kept at 0° C and 90% RH. *n* = 9 for the weight loss and respiration rate (three replications x three different measurements for each replication). *n* = 60 for the firmness (three replications x ten fruit x two different measurements for each fruit). * The scale ranges from 0 to 100 for very soft to very firm surfaces. Means in columns with the same letter do not differ according to Tukey’s test at *P* < 0.05

In the study examining the effects of post-harvest MeJA application on fruit quality in jujube, it was found that the weight loss in the fruits increased during the storage period and reached 1.4% in the fruits in the control group. The highest values ​​during storage were recorded in the control application. MeJA application was effective in reducing weight loss in fruit during storage. However, no difference in effect was observed between the MeJA application doses (Fig. 1). Similar results have been reported in previous studies. Asghari and Hasanlooe [17] stated that moisture loss from the fruit surface reduces fruit quality by causing cellular and membrane degradation, which in turn leads to earlier ageing and loss of market value. MeJA application limits water loss in fruit cells during this process, ensuring that they remain fresh, which delays fruit deterioration [12]. Thanks to its antioxidant properties, MeJA has been found to prevent membrane peroxidation by combating free radicals and thus reduce the rate of fruit deterioration [18]. Furthermore, Serna-Escolano et al. [19] noted that MeJA suppressed weight loss in lemons during long-term storage. The effect of MeJA, in addition to helping prevent fruit senescence and water loss, also allows the preservation of cellular integrity. Ezzat et al. [20] reported that MeJA works as an anti-senescence agent and prevents weight loss by preserving cellular structures. These findings are consistent with the study results. The effect of MeJA has been similarly observed in different fruit species such as mango [21], strawberry [17], apricot [20] and orange [22]. MeJA is thought to inhibit the respiration rate by slowing down the metabolic processes of the fruits and lead to stomatal closure [23]. As a results, the effect of MeJA on Jujube fruit is highly effective in reducing weight loss and maintaining fruit quality during storage period.

### Respiration rate

Fruit respiration rate is one of the most important biochemical parameters affecting quality and shelf life during the postharvest fruit storage process, especially in climacteric fruit species, the changes in respiration rate are considered as an indicator of the ripening process. During the period when the respiration rate increases, the fruit metabolism accelerates and the ripening process begins, which shortens the shelf life of the fruit. On the 15th day of storage, it was observed that the respiration rate in the control group remained at the same level as at the time of harvest. This situation shows that the fruits have adapted to the storage environment and their metabolic activities have not yet changed. However, the significant decrease in the respiration rate was observed in the MeJA-applied groups. On the 30th day of storage, an increase in respiration rate was observed in all applications. Although this increase indicates that the fruit has started to age and its metabolic activities have accelerated, the respiration rate remained at lower levels in the MeJA-treated fruits. In the control group, the respiration rate was measured at a high value of 18.5 CO_2_ kg^− 1^ s^− 1^ while this rate remained at lower levels in MeJA applications. This finding supports the fact that MeJA slows down the metabolic rate of the fruit, extending fruit quality and shelf life. At the end of storage, the decrease in respiration rate was observed in all applications. However, the lowest respiration rate was measured in the fruits treated with 0.4 mM MeJA This finding shows that 0.4 mM MeJA most effectively limits fruit respiration, preserving fruit quality in the best way and extending shelf life (Fig. 1). Islam et al. [24] stated that the respiration rate increases in proportion to the storage time and this increase occurs during the period when the carbon dioxide rate increases in climacteric fruits. MeJA slowing down the metabolic rate contributes to preserving fruit quality and extending storage time [10]. Furthermore, this finding is in line with previous studies that MeJA improves post-harvest fruit quality, increases antioxidant levels and reduces cold damage [21, 25]. In particular, it is confirmed by many studies in the literature that the rate of aging slows down in groups where methyl jasmonate is applied during the fruit ripening process, thus preserving fruit quality and extending shelf life [18, 26]. The studies on the mechanism of action of MeJA show that this compound improves fruit quality by increasing the biosynthesis of phenolic compounds and regulating antioxidant systems [10, 25]. In particular, MeJA application is reported to reduce the effects of cold damage and oxidative stress by increasing the activities of antioxidant enzymes [27]. This effect helps preserve fruit quality by keeping the respiration rate at low levels.

### Fruit firmness

Fruit firmness is an important factor determining fruit quality and storage potential [28, 29]. In this study, a decrease in flesh firmness of jujube fruits occurred with the advancement of postharvest storage period. It was determined that the fruits of the control application were softer during the storage period. MeJA application had a positive effect on preserving the flesh firmness of the fruit. However, there was no difference in fruit flesh firmness between the MeJA application concentrations (Fig. 1). This decrease in fruit firmness parallels the ripening process of the fruit and can be explained by the hydrolysis of pectins in the cell walls and the degradation of structural components such as cellulose and hemicellulose [24, 30, 31]. This suggests that from the beginning of the storage process, the turgor pressure of the fruit decreases and structural changes in the cell wall cause a loss of firmness. MeJA application helped maintain higher fruit flesh firmness compared to the control group. This finding demonstrates the potential of MeJA to maintain postharvest fruit quality (Fig. 1). The effects of MeJA are associated with its function as a defense mechanism against abiotic stresses in plants. MeJA application can regulate the activity of cell wall-associated enzymes such as pectin methylesterase (PME) and phenylalanine ammonium lyase (PAL) by enhancing cell wall stability, which may help maintain fruit firmness [12, 32]. Additionally, MeJA has been reported to increase cell wall strength by stabilizing Ca^2+^ levels in the cell wall, and this mechanism is effective in maintaining fruit firmness [33, 34]. The fact that the effect of MeJA does not change depending on the dose is also supported by the fact that there is no significant difference between the 0.2 mM and 0.4 mM concentrations. This shows that both concentrations are similarly effective and the effect of MeJA reaches the maximum level up to a certain dose [35]. This finding suggests that the effectiveness of MeJA may remain constant beyond concentration and that higher doses may not provide additional benefits. This supports previous studies showing that the effects of MeJA may vary depending on plant species and fruit variety. Similarly, there are conflicting findings in the literature regarding the different effects of MeJA on different fruit species. For example, Meng et al. [33] reported that MeJA application in peach, apple and apricot delayed the softening process by increasing fruit firmness, while some studies did not observe a significant effect on firmness in tropical fruits such as mango and papaya [21]. These differences indicate that the effect of MeJA on fruit firmness may vary depending on the fruit type, environmental conditions and applied concentration. In a study conducted by Shah et al. [36], it was stated that MeJA application delayed the loss of fruit firmness in raspberries, which could be explained by the decrease in enzyme activities that prevent cell wall destruction and the increase in Ca^2+^ levels.

### Fruit color (L*, chroma and hue)

**Fig. 2:**
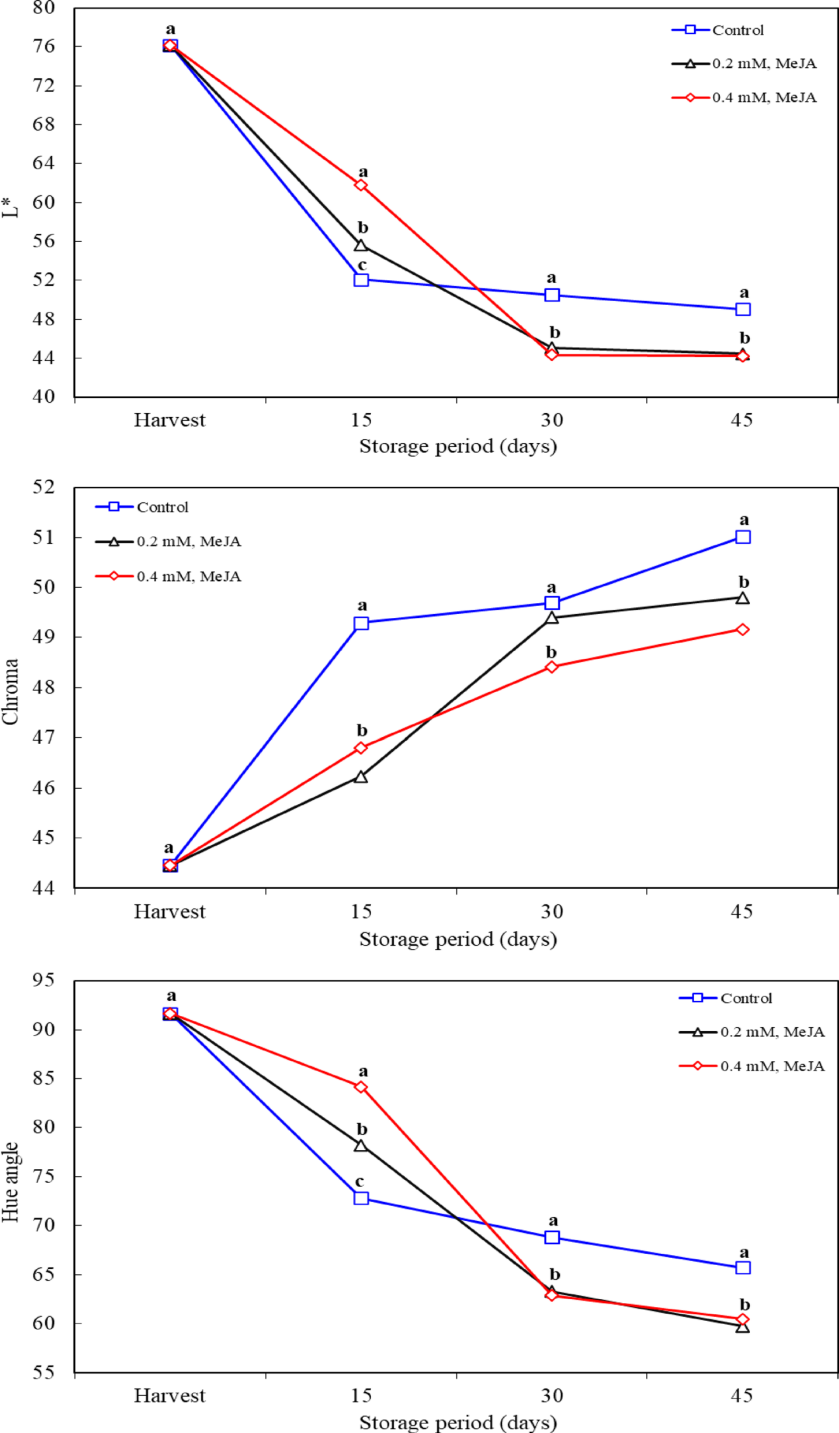
Effects of methyl jasmonate treatments on L*, chroma and hue angle of jujube fruit (*Ziziphus jujuba*) kept at 0° C and 90% RH. *n* = 60 for the L*, chroma and hue angle (three replications x ten fruit x two different measurements for each fruit). Means in columns with the same letter do not differ according to Tukey’s test at *P* < 0.05

Fruit color is one of the most important quality traits that directly affects consumer acceptance. Especially under cold storage conditions, fruit color often changes with the ripening process, and this process can lead to undesirable visual changes on the fruit. In this study, the effects of 0.2 mM and 0.4 mM MeJA applied to jujube fruit on fruit color values ​​were investigated. The findings of the study show that MeJA helps preserve color values ​​by slowing down fruit ripening, thus ensuring that fruit quality is kept high for a long time. As the storage period progressed, a general decrease in L* value was observed. This indicates that the ripening fruit surface loses its shine and takes on a paler appearance, reflecting the aging process of the fruit. On the 15th day of storage, the highest L value was recorded with the application of 0.4 mM MeJA. This finding suggests that MeJA slows down fruit ripening, helping to maintain brightness for a longer period. Similarly, the decrease in hue is often associated with yellowing or browning as the fruit ripens. The decrease in hue was observed on the 30th and 45th days of storage, but it can be said that MeJA applications slowed down this process and the fruit color remained more vibrant and fresh. No difference in hue was observed between 0.2 mM and 0.4 mM MeJA applications, suggesting that both concentrations were similarly effective and that the capacity of MeJA to delay changes in fruit color was independent of the dose. Chroma value refers to the color saturation of the fruit, and as the fruit ages, the chroma value generally increases. It was observed that the chroma value of the fruits in the control group increased during storage, but the chroma value remained at lower levels in MeJA applications (Fig. 2). This finding suggests that MeJA slows down fruit senescence and ripening, preventing premature increase in color saturation, thus maintaining high fruit quality for a longer period of time. This finding is consistent with the effect of MeJA in delaying fruit senescence and preserving color change [36, 37]. MeJA is a compound that activates stress coping mechanisms in plants and is particularly known for its regulatory effect on the fruit ripening process. It has been frequently reported in the literature that MeJA slows down fruit ripening by regulating ethylene biosynthesis, thus delaying color changes and preserving fruit quality [35]. In addition, MeJA stabilizes Ca^2+^ levels in the cell wall, allowing pectins to bind with Ca^2+^ and slowing down color changes on the fruit surface [34]. These interactions of MeJA play an important role in maintaining fruit quality during post-harvest period. There are many studies in the literature where MeJA improves color properties in various fruits. Aslantürk et al. [38] reported that MeJA affects color change in apricot. Shah et al. [36] and Muengkaew et al. [37] reported that MeJA preserves fruit color, delays anthocyanin degradation and increases flavonoid levels. Moreover, the studies such as Deshi et al. [39] and He et al. [40] indicated that MeJA applications effectively delay browning of fruits such as litchi, apricot, and rosehip. Such findings support that MeJA is an effective agent in inhibiting fruit senescence and discoloration in general.

### Soluble solids content (SSC) and titratable acidity

**Fig. 3:**
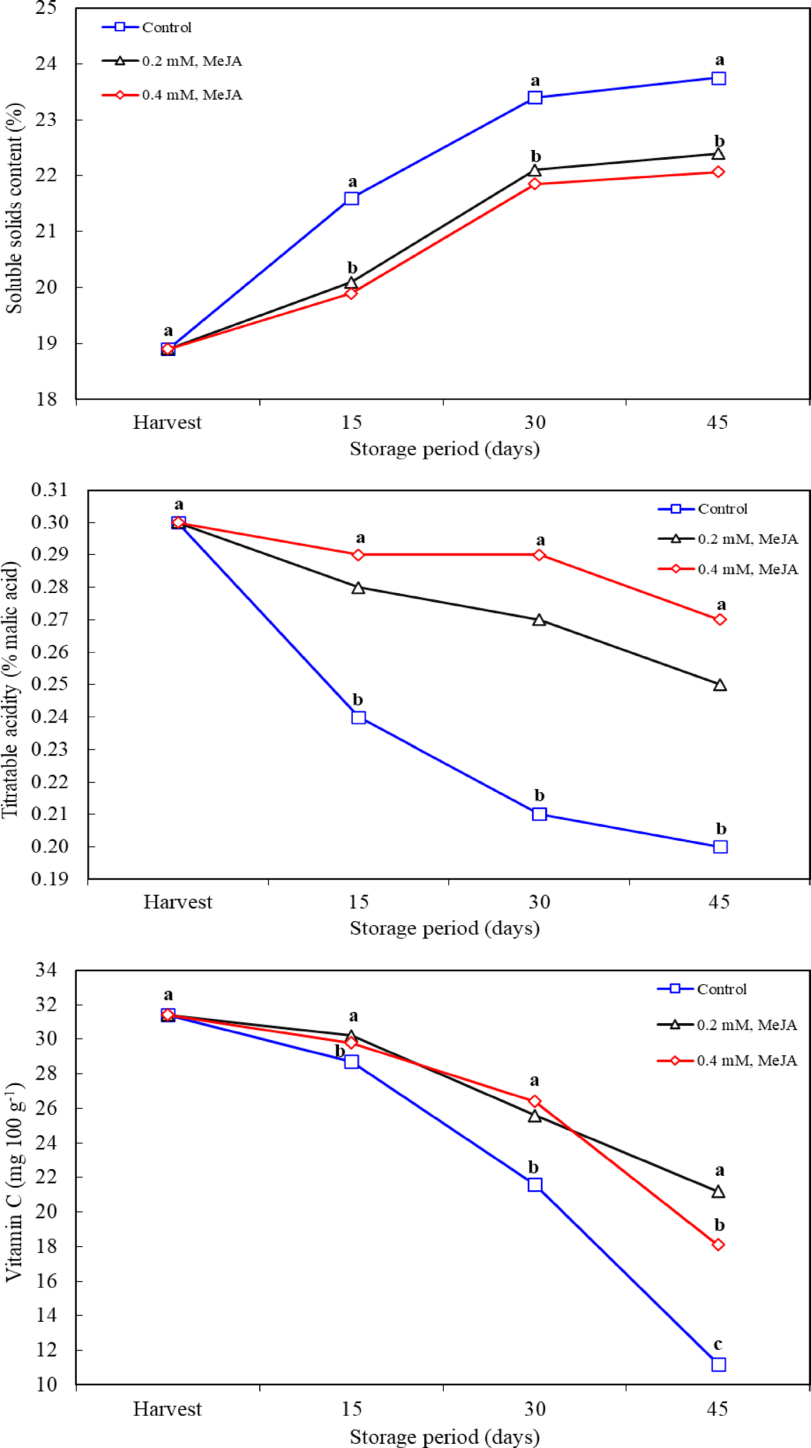
Effects of methyl jasmonate treatments on soluble solids content, titratable acidity and vitamin C of jujube fruit (*Ziziphus jujuba*) kept at 0° C and 90% RH. *n* = 9 for the soluble solids content, titratable acidity and vitamin C (three replications x three different measurements for each replication). Means in columns with the same letter do not differ according to Tukey’s test at *P* < 0.05

Biochemical properties such as SSC and titratable acidity are the main parameters determining fruit quality and directly affect consumer perception with fruit taste profile, nutritional values. The effect of MeJA, as a growth regulator that slows down fruit ripening, may play an important role in maintaining fruit quality, especially in postharvest processes. SSC determines the flavor intensity of the fruit as an indicator of sugars, acidic compounds and other dissolved solids in the fruit content. It was found that the SSC rate in the fruits increased during the storage period. The highest values ​​during storage were recorded in the control application. MeJA application was effective in maintaining SSC in fruit during storage. However, no difference in effect was observed between the MeJA application doses. According to the study findings, the increase of SSC during storage period is a natural result of fruit ripening (Fig. 3). This is associated with the hydrolysis of polysaccharides into simple sugars and the accumulation of sugars in the fruit [41]. MeJA application prevented this increase, the lower SSC values ​​were shown in MeJA-treated fruits. It is thought that MeJA slows down fruit ripening by inhibiting ethylene biosynthesis, thus delaying sugar accumulation [42]. In various studies, MeJA application has been reported to increase SSC value. For example, MeJA application has been reported to have positive effects on SSC in ‘Kinnow’ mandarin by Baswal et al. [12] and in plum by Kucuker and Ozturk [43]. However, in this study, SSC remained at lower levels with MeJA application, which contradicts the findings in other studies. However, the decrease in SSC by MeJA application, which slows down fruit ripening by inhibiting the effect of ethylene, is parallel to the effect of delaying senescence and metabolic activities observed in studies such as Zapata et al. [44] and Serna-Escolano et al. [19].

Titratable acidity determines the acidic content of the fruit and affects the acidic intensity of the fruit’s taste. As the storage period progresses, the acidity rate in the fruit decreases as a natural result of ripening. However, this decrease in acidity was lower in MeJA-treated fruits and was not significant in measurements made during storage between MeJA application doses (Fig. 3). This suggests that MeJA helps maintain acidic compounds for a longer time by slowing down fruit ripening. MeJA’s inhibition of the action of ethylene slows down the metabolism of organic acids and preserves acidity [45]. Similarly, González-Aguilar et al. [21] and Baswal et al. [12] reported that MeJA application increased fruit acidity and maintained this effect. As the storage period progresses, the fruits in the control group ripen more and acidic compounds are metabolized in this process, thus decreasing titratable acidity levels (Fig. 3). Similarly, in the studies of Islam et al. [24] and Chen et al. [46], it was found that the acidity content decreased during cold storage, but the fruits treated with MeJA retained acidity for a longer time. These findings confirm that MeJA is effective in slowing down fruit ripening and preserving acidity. The mechanism of action of MeJA is mainly based on slowing down fruit ripening. MeJA delays fruit ripening by inhibiting the biosynthesis of ethylene, which allows the fruit to retain its taste and acidity properties for a longer period. The studies such as Shah et al. [36], Serna-Escolano et al. [19] and Zapata et al. [46], reported that MeJA application delayed fruit senescence and was consequently effective in maintaining both SSC and titratable acidity. The discrepancies between study results may be due to differences in species, cultivars, application doses, and timing. In studies such as Baswal et al. [12] and Kucuker and Ozturk [43], it was reported that MeJA increased SSC while in studies such as Zapata et al. [44] and Serna-Escolano et al. [19], the effect of MeJA seems to be to slow down fruit ripening and maintain acidity.

### Vitamin C

In the evaluation of the vitamin C content of the study, a significant relationship was observed between storage time and loss of vitamin C. As storage time increased, a decrease in vitamin C level occurred in all treatments, but MeJA treatment was found to significantly reduce this loss. In particular, the highest vitamin C value was measured in fruits treated with 0.2 mM MeJA on the 45th day, the clearly demonstrating the effect of MeJA on maintaining the vitamin C content. No significant difference was observed between the application doses of MeJA on the 15th and 30th days, but the effects of MeJA were clearly evident on the 45th day of storage (Fig. 3). Vitamin C is critical to the nutritional value of fruits and vegetables and can be rapidly reduced by factors such as oxidative stress, enzymatic oxidation and water loss during the postharvest process. Gao et al. [1] emphasized the rich vitamin C content of jujube fruit and emphasized the importance of preserving this content. Zhou et al. [47] stated that the activity of ascorbic acid oxidase and phenol oxidase enzymes increased during the postharvest fruit ripening process, which led to vitamin C loss. These findings may indicate that the reduction of vitamin C loss in MeJA applied-fruits is related to the inhibition of enzymatic oxidation processes. This mechanism of action of MeJA is also consistent with other similar studies in the literature. Shah et al. [36] reported that MeJA application helped maintain higher vitamin C levels in fruits such as raspberries under cold storage conditions. This may be related to the delaying effect of MeJA on oxidation reactions. In addition, the study conducted by Mustafa et al. [48] reported that antioxidant enzyme activities increased in cold-stored carambola fruit. This increase may be related to the delay in the ripening and aging process of the fruit. This supports the potential of MeJA to increase vitamin C levels, particularly through upregulation of genes involved in ascorbic acid biosynthesis [23]. This mechanism could also be explained by MeJA’s regulation of metabolic activities such as dehydroascorbatese, enzymes that inhibit the oxidation of ascorbic acid. Cai et al. [49] on loquat, Muengkaew et al. [37] on mango, Baswal et al. [12] on mandarin and Wang et al. [50] on blueberry also reported similar effects of MeJA. These findings suggest the ability of MeJA to maintain vitamin C content in a wide range of fruit types and that this could potentially be an effective strategy to improve postharvest quality.

### Total phenolics and total flavonoids

**Fig. 4:**
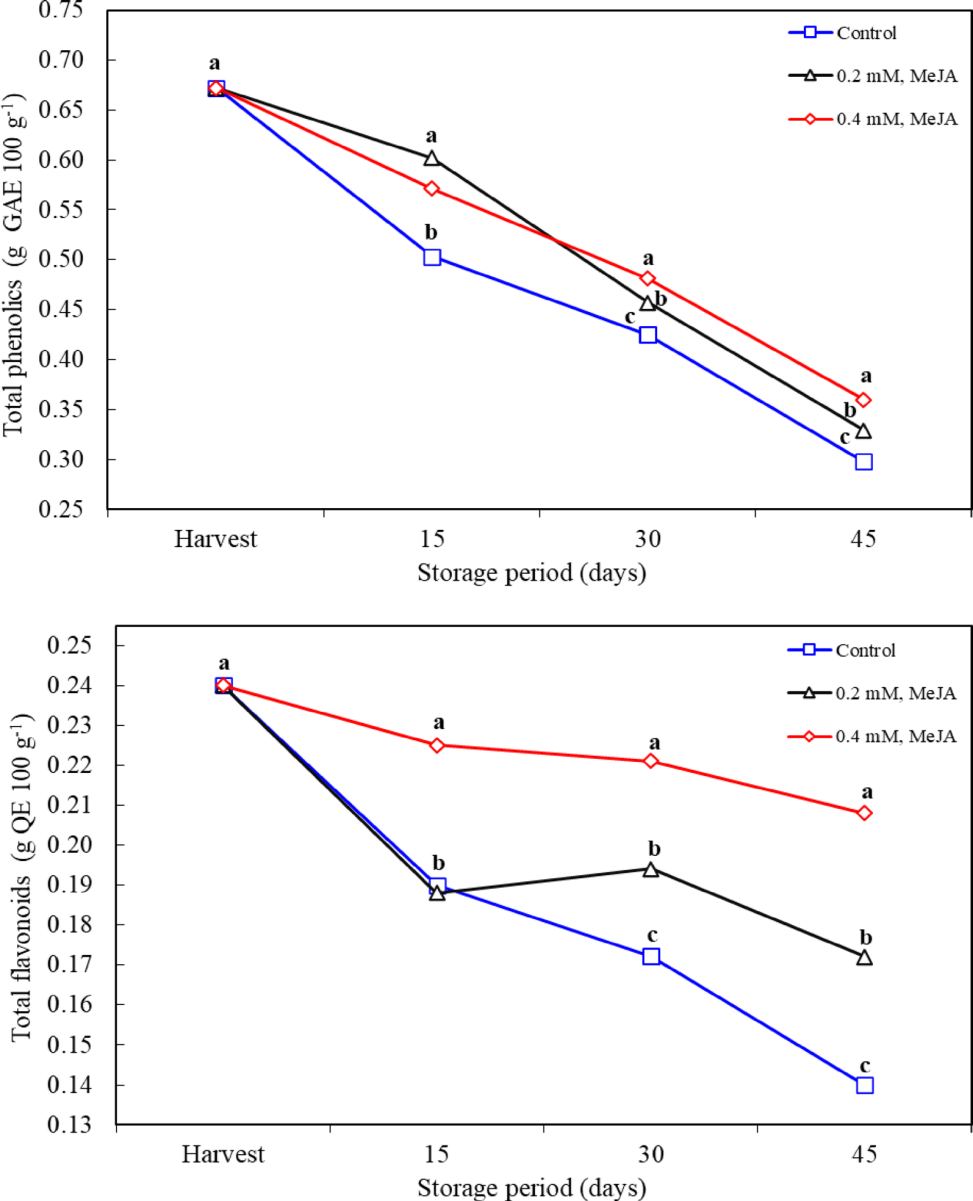
Effects of methyl jasmonate treatments on total phenolics and total flavonoids of jujube fruit (*Ziziphus jujuba*) kept at 0° C and 90% RH. *n* = 9 for the for the total phenolics and total flavonoids (three replications x three different measurements for each replication). Means in columns with the same letter do not differ according to Tukey’s test at *P* < 0.05

Phenolic compounds and flavonoids are important antioxidant components of fruits and improve fruit quality as well as increase the ability of plants to combat stress [51]. The levels of these compounds can be affected by extrinsic factors such as genetic factors, environmental conditions, and storage times. In particular, it has been reported that as the cold storage period increases, phenolic compounds in the fruit deteriorate and their concentrations decrease [4]. Similarly, Islam et al. [24] stated that increasing the storage period decreases the phenolic concentration. This is due to the oxidation of phenolic compounds. The decrease in total phenolic and flavonoid ratios was observed in all applications during the storage period. However, MeJA application was effective in maintainig the phenolic and flavonoid content. While there was no significant difference between different application doses on the 15th day of storage, it was observed that 0.4 mM MeJA dose was more effective on the 30th and 45th days. These findings suggest that MeJA offers an important strategy for maintaining total phenolic and flavonoid content during postharvest storage. The effect of MeJA in this process is remarkable (Fig. 4). MeJA contributes to the preservation of phenolic content, especially by increasing the activity of phenylalanine ammonia-lyase (PAL), which plays an important role in the biosynthesis of phenolic compounds [21, 52]. In addition, MeJA inhibits the oxidation of phenolic compounds by suppressing polyphenol oxidase (PPO) activity, which may help maintain the antioxidant capacity of fruits [17]. Shah et al. [36] reported that flavonoid and anthocyanin levels were higher in MeJA-applied raspberries than in the control group. Habibi et al. [53] reported that MeJA preserved the phenolic content by inhibiting PPO activity in oranges. It is also important to note that phenolic compounds are important antioxidants that initiate stress-related defense mechanisms in plants [54]. Upregulation of the biosynthesis of these compounds by MeJA may help improve fruit quality and prevent oxidative damage during storage. These findings indicate that MeJA is an effective tool in maintaining both phenolic compounds and flavonoids. In particular, the studies such as Shah et al. [36] and Shafiq et al. [55] confirm that MeJA exerts positive effects on flavonoid and anthocyanin content. Among the mechanisms of action of MeJA, regulation of phenylalanine ammonia-lyase (PAL) and polyphenol oxidase (PPO) activities is at the forefront. PAL is the rate-limiting enzyme of the biosynthesis of phenolic compounds, and MeJA appears to promote the production of phenolic compounds by increasing the activity of this enzyme [18, 21]. In addition, inhibiting PPO helps maintain fruit quality for a longer period by preventing the oxidation of phenolic compounds [36]. Studies show that MeJA causes similar effects in different fruit species. For example, Wang et al. [50] noted that MeJA increased the flavonoid content in blueberry, Baswal et al. [12] in mandarine, and Garcia-Pastor et al. [56] in pomegranate. In addition, MeJA has been reported to increase the phenolic and flavonoid content in fruits such as mango [37].

### Antioxidant activity

**Fig. 5:**
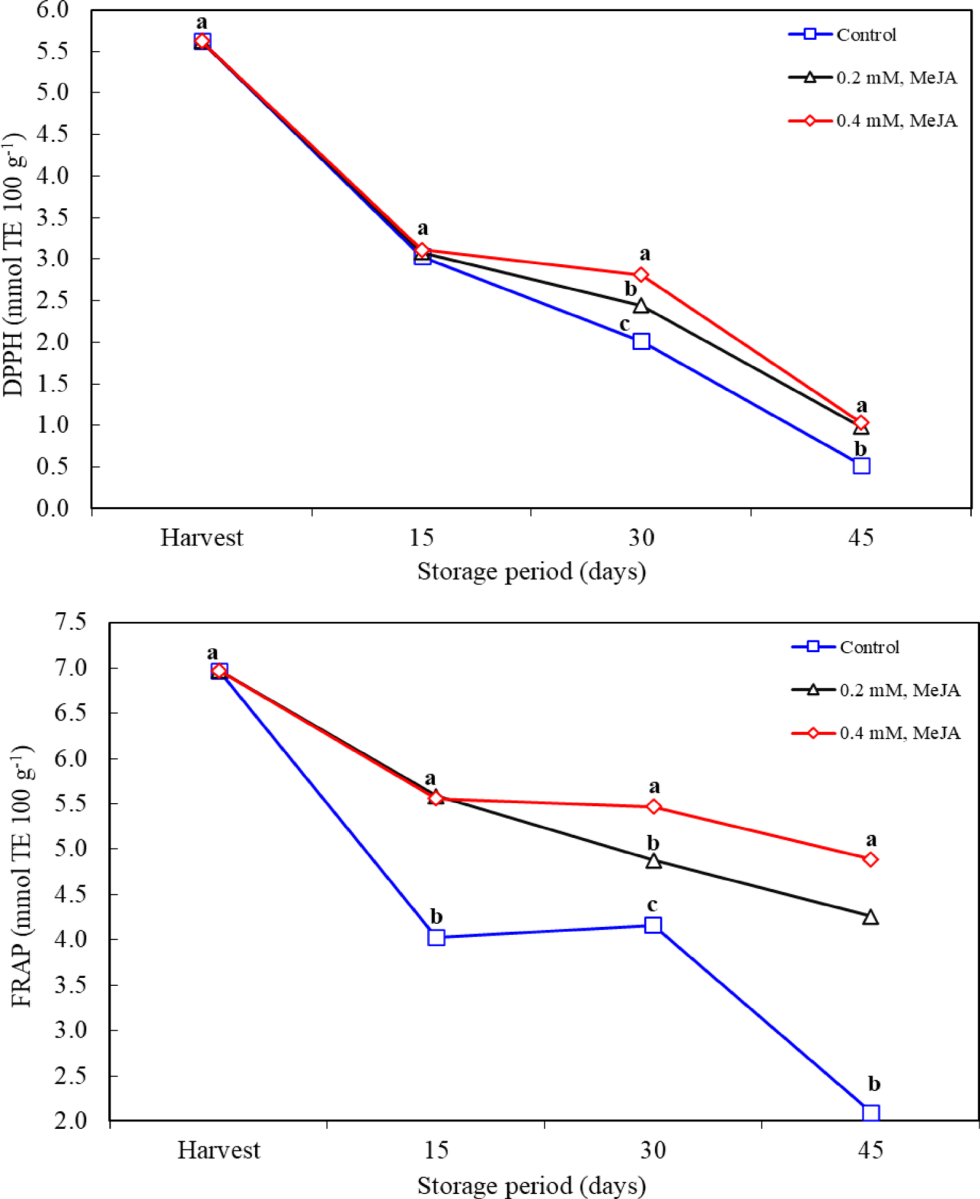
Effects of methyl jasmonate treatments on antioxidant activity (in DPPH and FRAP assays) of jujube fruit (*Ziziphus jujuba*) kept at 0° C and 90% RH. *n* = 9 for the antioxidant activity for DPPH and FRAP (three replications x three different measurements for each replication). Means in columns with the same letter do not differ according to Tukey’s test at *P* < 0.05.

There was a decrease in antioxidant activity determined as FRAP and DPPH in all applications depending on the storage period. The lowest antioxidant activity values ​​during storage were recorded in the control application. MeJA application was effective in maintaining the antioxidant activity content. There was no difference between the application doses on the 15th and 45th days of storage, but it was observed that MeJA application doses were effective on the 30th day of storage and 0.4 mM MeJA application was more effective (Fig. 5). Prolonged storage period leads to deterioration of fruit quality, especially the decrease of phenolic compounds, flavonoids and other antioxidant compounds [4, 24]. The findings of this study suggest that MeJA application helps maintain these compounds. In previous studies, the ability of MeJA to increase postharvest antioxidant capacity was supported by the elevation of antioxidant enzyme activity in fruits and vegetables. In particular, the increased activities of enzymes such as SOD, PPO, CAT indicate the effectiveness of MeJA in scavenging reactive oxygen species (ROS) [57]. The effect of MeJA is not limited to the increase of phenolic compounds. There is strong evidence that MeJA prevents ROS accumulation by increasing enzymatic activity in fruit cells and helps increase antioxidant capacity. These effects are associated with the increase in the levels of antioxidant compounds, especially ascorbic acid, glutathione, and flavonoids [58, 59]. These findings are in line with the data in our study, because the main mechanism behind the preservation of antioxidant activity during MeJA application is the reduction of oxidative stress and the increase of the stability of phenolic compounds. Many studies on the effect of MeJA on postharvest antioxidant activity have shown similar results in different fruit species. For example, MeJA application has been shown to increase antioxidant capacity in blood oranges, blueberries and other fruits [59]. In particular, Habibi et al. [53] reported that MeJA preserved antioxidant activities in blood oranges during 150 days of storage. As in this study, the ability of MeJA to preserve antioxidant components during storage may vary depending on the fruit species. Furthermore, the effect of MeJA is also related to storage temperatures. For example, studies at low temperatures (0–6 °C) have shown the efficacy of MeJA in enhancing antioxidant capacity, but studies at 20 °C indicated that MeJA showed greater effects [60]. This suggests that the effect of MeJA may become more pronounced under different environmental conditions. MeJA is a plant growth regulator that can stimulate stress-related defense mechanisms in plants. The studies have shown that MeJA stimulates the biosynthesis of phenolic compounds by increasing the activities of phenylalanine ammonia lyase (PAL) and PPO enzymes [15, 52]. The regulatory effect of MeJA on these biosynthesis pathways plays a critical role in improving fruit quality. In particular, MeJA application prevents the oxidation of phenolic compounds, making fruits more resistant to the effects of oxidative stress during storage [32]. These findings are consistent with the results of this study; MeJA application was effective in preserving antioxidant activities and prevented the degradation of antioxidant components.

## Conclussion

This study shows that methyl jasmonate (MeJA) application is an important and effective method in preserving the quality and biochemical properties of jujube fruits after harvest. The findings show that MeJA application slows down the ripening process of the fruit and preserves the color, texture and nutritional content during storage. In MeJA treated fruits, lower weight loss, slower respiration rate and better preservation of SSC, titratable acidity and vitamin C levels were observed compared to the control group. In addition, MeJA was also effective in preserving phenolics and flavonoids that play an important role in the antioxidant capacity of fruits. Although both 0.2 mM and 0.4 mM MeJA doses were beneficial, it was determined that the higher dose was slightly more effective in terms of preserving quality. These results reveal that MeJA can be used as a current and applicable postharvest preservation strategy with the potential to extend the shelf life and increase the commercial value of jujube fruits and make an important contribution to the studies in this field.

## Supplementary Information

Below is the link to the electronic supplementary material.

Supplementary Material 1

## Data Availability

All data generated or analyzed during this study are included in this published article.

## Abbreviations

CAT: Catalase
DPPH: 2,2-diphenyl-1-picrylhydrazyl
FRAP: Ferric reducing antioxidant power
FW: Fresh weight
GAE: Gallic acid equivalent
MeJA: Methyl jasmonate
PAL: Phenylalanine ammonia-lyase
PPO: Polyphenol oxidase
ROS: Reactive oxygen species
SOD: Superoxide dismutase
SSC: Soluble solids content
QE: Quercetin equivalent
