# Supplementary material for: Impacts of methyl jasmonate treatments on quality properties and bioactive compounds of jujube fruit (Ziziphus jujuba) during cold storage
Source: BMC Plant Biol. 2025 Aug 11;25:1064. doi: 10.1186/s12870-025-07140-2 (PMC12337489; doi:10.1186/s12870-025-07140-2)
Supplement: Supplementary file 1 — Supplementary Material 1 [file 12870_2025_7140_MOESM1_ESM.pdf]

Technical Check of your submission

Dear Editor

The requested technical correction has been made and added to the relevant part of the text and highlighted in red.

Thank you very much for your help.

Comment 1: Please provide a Funding Declaration in the manuscript. If there was no Funding, please state so.

Response: A Declaration in the manuscript was expressed as "Funding: Not applicable."

Assoc. Prof. Dr. Erdal Aglar

Munzur University, Tunceli-Türkye

Email: erdalaglar@gmail.com

Phone: +90.432.2251056

Fax: +90.432.2251056
